# Genes and environment in multiple sclerosis: Impact of temporal changes in the sex ratio on recurrence risks

**DOI:** 10.1177/13524585211020221

**Published:** 2021-06-08

**Authors:** A Dessa Sadovnick, Irene M Yee, Maria Criscuoli, Gabriele C DeLuca

**Affiliations:** Department of Medical Genetics, University of British Columbia, Vancouver, BC, Canada/Division of Neurology, Department of Medicine, University of British Columbia, Vancouver, BC, Canada/UBC Hospital, University of British Columbia, Vancouver, BC, Canada; Department of Medical Genetics, University of British Columbia, Vancouver, BC, Canada; Department of Medical Genetics, University of British Columbia, Vancouver, BC, Canada; Nuffield Department of Clinical Neurosciences, John Radcliffe Hospital, Oxford, UK

**Keywords:** Multiple sclerosis, recurrence risks, sex ratio, environment, genetics

## Abstract

**Objective:**

To evaluate the impact of temporal increase of female to male (F:M) sex ratio for persons with multiple sclerosis (MS) on the familial risk (empiric recurrence risks or RRs) for biological relatives of affected individuals.

**Methods:**

Detailed family histories were systematically obtained from people with MS attending the University of British Columbia Hospital MS Clinic. The study cohort was born in 1970 or more recently. Data were collected from 1 September 2015 to 31 January 2019. The study was designed to allow only one proband per family. Age-corrected RRs for biological relatives of probands were calculated based on a modification of the maximum-likelihood approach.

**Results:**

Data analyses were possible for 746 unique probands (531 females; 215 males) and 19,585 of their biological relatives. RRs were temporally impacted.

**Conclusion:**

Both genetic sharing and environmental factors are important in determining RRs. It appears that there is an increase in MS risk due to environmental factors in later life (i.e. not shared family environment). Environmental exposures in genetically predisposed individuals might be driving the MS risk. The increase in F:M ratio of RRs for sisters/brothers of female probands over time is likely due to environmental differences.

## Introduction

Multiple sclerosis (MS), a chronic inflammatory demyelinating disorder of the central nervous system, is the most common cause of neurological disability among young adults. The etiology of MS is unclear but genes, environment, and their interactions are believed to be important and key contributors to MS in general as well as to the familial aggregation of the disease.^1^ Empiric recurrence risks (RRs) are used in genetic counseling for common complex disorders such as MS to provide information for “at-risk” biological relatives.^2,3^ RRs vary by sex of the proband, sex of the “at-risk” relative, the number of affected relatives in the family, and the degree of relatedness to the proband.

Population-based empiric RRs for MS were presented in the late 1980s.^4^ The initial RR data^4^ were based on a birth cohort of consecutive people with MS born well before 1970 who attended the University of British Columbia (UBC) Hospital MS Clinic (hereafter referred to as the “MS Clinic”). At that time, there was close to a 1:1 female to male sex ratio (F:M ratio) among affected individuals within multi-case (multiplex) families compared to an approximate 1.4:1 sex ratio for the general population. More recent studies from various regions including Canada,^5^ Denmark,^6^ Sweden,^7^ Norway,^8^ and Crete^9^ have all shown an increasing F:M ratio for MS prevalence over time. This study revisits the topic of RRs in biological relatives of people with MS attending the MS Clinic and examines the temporal change, if any, in the F:M ratio for RRs.

Here, we provide evidence that RRs are influenced by environmental factors, with genetic predisposition to MS only explaining part of the disease risk for the general population as well as the familial aggregation. MS heritability is estimated at 50%^10^ and individual genetic variants additively explain 22.4% of the liability for MS (i.e. 22%/50% = 44% of heritability).^11–13^ The remainder of heritability is explained by complex genetic, epigenetic, and genetic/environment interactions.

## Methods

“The mandate of the UBC Hospital MS Clinic has been a multidisciplinary team approach with the end goal of finding the cause and cure of MS through patient management and education, research, and teaching.”^14^

Between 1 September 2015 and 31 January 2019, detailed family histories were collected from patients attending the MS Clinic who were born in 1970 or more recently and for whom appropriate informed consent had been obtained. Consent included collection and storage of de-identified demographic, clinic, laboratory, and family history information of people with MS in the MS Clinic Research Databases and permission to recontact for future research studies. There is also a section in the consent form requesting permission to contact other family members for an accurate completion of the family history, confirmation of MS diagnosis, and demographic data. MS Clinic diagnoses initially used the Poser criteria^15^ and, since their introduction, the McDonald and revised McDonald criteria, the most recent of which was published in 2018.^16^ Although successive versions of diagnostic criteria have differed in emphasis, all have required dissemination of disease in space (DIS) and time (DIT) documented by either clinical, paraclinical, or laboratory criteria.

MS Clinic neurologists annually review the medical records of all patients and change diagnoses as appropriate when additional clinical, imaging or laboratory results become available. With the revised McDonald criteria, the diagnosis of MS has been made more often and earlier for both men and women.^17^ It is unlikely that the revised criteria influence the change in the F:M ratio.

The study was designed to allow only one proband per family, defined as the first biological family member with MS born in 1970 or more recently ascertained through the MS Clinic. Care was thus taken to identify MS Clinic attendees who were biologically related and separately agreed to participate in this study. Family information was collected through a structured, standardized telephone interview, following the methodology for the earlier RR study.^4^ The diagnosis of MS was carefully documented in biological relatives of probands. MS status of the affected family member was confirmed by physician and/or hospital records where possible or validated by other family members. Accuracy of this method was validated by natural history studies.^18^

This study was approved by the UBC Clinical Research Ethics Board (UBC CREB) and the Vancouver Coastal Health Research Institute (VCHRI).

### Statistical analysis

Crude RRs for MS were calculated for different categories of relative by dividing the number of affected relatives by the total number of relatives. Age-corrected empiric RRs for the relatives were calculated based on a modification of the maximum-likelihood approach.^19^ Age-correction takes into account the fact that certain relatives may not have reached the age of maximum risk. Lifetime recurrence risks for each category of relative can be estimated by dividing the number of affected relatives by the adjusted number of such relatives at risk. The maximum-likelihood risk estimation requires the use of a prior age-of-onset distribution. The prior cumulative age-of-onset distribution was estimated from the 744 probands with known age of onset. The distribution varied from 0% at age 3 years to 100% at age 43 years, the oldest age at which the first symptoms of MS have manifested in this group of probands. Under this approach, the estimate of RR and its error are reasonably robust with respect to the form of age-of-onset distribution used.^19^ Comparisons of RRs were assessed with likelihood ratio test (LRT) statistic, which has an approximate chi-square distribution with one degree of freedom.^19^ All age-adjusted RRs are presented with 95% confidence intervals (CIs) and results of the LRT are given.

Under the assumption that the two study samples (1988^4^ and this study) were independent, a *z*-score test was used to compare the sex ratios. The one-sided *z*-test with a level of significance of 5% was used to compare the natural logarithm of sex ratios between the two studies to investigate the direction of change in sex ratio over time. A *p*-value of < 0.05 was considered statistically significant. These sex ratios are presented with 95% CIs.

Our focus was on the RRs estimated for biological parents, siblings, aunts/uncles, and first cousins. These biological relatives were selected for in-depth analyses as they require less age adjustment than would children and nieces/nephews of probands. Unfortunately, information on first cousins was incomplete (geographic distance, family dynamics, etc.) so this group could not be as thoroughly investigated as we had hoped. Thus, the RRs data reported here are limited to parents, aunts/uncles, and siblings.

It is important that comparing sex ratios in birth cohorts who have passed through most, if not all, of their lifetime period of disease risk will eliminate the problems imposed by differential ages of symptom onset by sex, and by incomplete ascertainment of cases with later symptom onset, an extra caution when using the maximum-likelihood approach.^19^ Parents and aunts/uncles are more reflective of the birth cohorts used in our previous RR studies in BC.^4^ In contrast, siblings are more reflective of the birth cohort for this study with respect to environmental exposures that may have changed compared to those for previous generations (e.g. parents, aunts/uncles).

## Results

**Figure 1.**
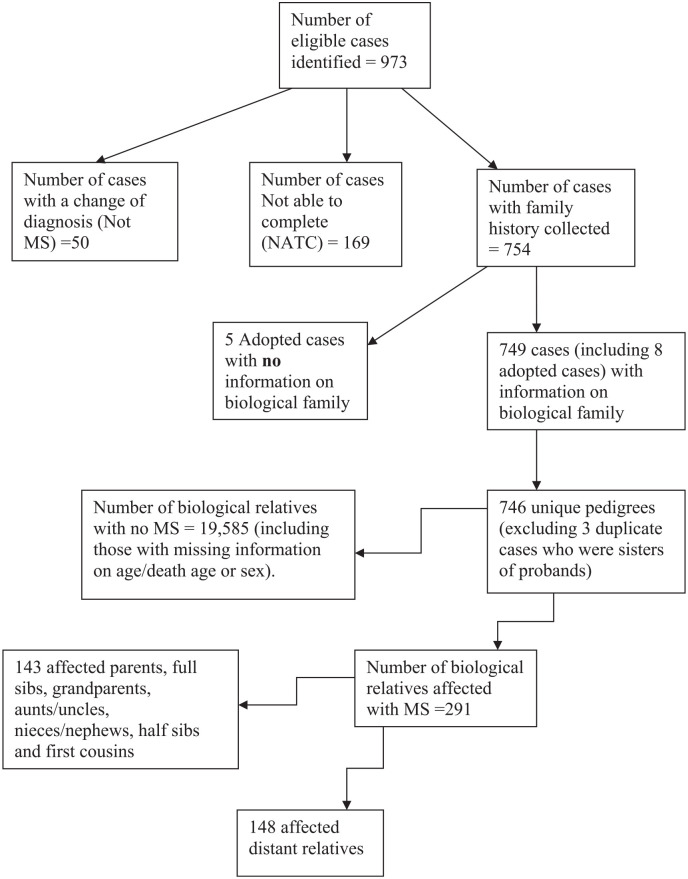
Flow chart on data collection.

A total of 973 eligible people with MS were identified between 1 September 2015 and 31 January 2019 at the MS Clinic. Figure 1 is a flow chart on data collection for the study. Fifty cases were excluded due to a change of diagnosis to “Not MS.” Pedigrees were thus collected from 754 people with MS (537 females and 217 males) of whom five patients were adopted with no information on their biological family. Three additional individuals had a sister who was also a MS Clinic patient meeting this study entry criteria. Thus, of these 754 potential probands, we were able to include a total of 746 unique probands (531 females; 215 males) with known family history information (see Figure 1) in the statistical analyses.

Basic demographic data for the 746 probands are given in Table 1 as are the number of relatives for whom information was available. There was no difference between family sizes reported by female and male probands (chi-square statistics = 4.04; df = 4; *p* = 0.40).

**Table 1.** Characteristics of 746 probands.

Year of birth	Probands		
	Female	Male	Total
1970–1974	208 (39.17%)	68 (31.63%)	276 (37.00%)
1975–1979	142 (26.74%)	65 (30.23%)	207 (27.75%)
1980–1984	106 (19.96%)	52 (24.19%)	158 (21.18%)
⩾ 1985	75 (14.12%)	30 (13.95%)	105 (14.08%)
Total	531 (100.00%)	215 (100.00%)	746 (100.00%)
Diagnosis of MS			
Definite MS	6 (1.13%)	4 (1.86%)	10 (1.34%)
Primary progressive MS	8 (1.51%)	9 (4.19%)	17 (2.28%)
Probable MS	2 (0.38%)	0 (0.00%)	2 (0.27%)
Relapsing remitting MS	499 (93.97%)	190 (88.37%)	689 (92.36%)
Secondary progressive MS	16 (3.01%)	12 (5.58%)	28 (3.75%)
Mean age at onset (SD) by birth cohort			
1970–1974	30.57 (6.39)	30.66 (5.77)	30.59 (6.23)
1975–1979	27.06 (5.33)	27.83 (5.09)	27.30 (5.26)
1980–1984	23.63 (5.08)	24.81 (4.09)	24.02 (4.80)
⩾ 1985	20.72 (4.36)	20.93 (4.16)	20.78 (4.29)
Overall	26.85 (6.64)^a^	27.03 (5.94)^b^	26.90 (6.45)^c^
Number of probands with at least one affected relatives^d^	91/531 (17.14%)	25/215 (11.63%)	116/746 (15.55%)
Number of relatives^d^	11,561	4394	15,955
Family size reported by proband			
⩽ 10	69 (12.99%)	30 (13.95%)	99 (13.27%)
11–20	204 (38.42%)	83 (38.60%)	287 (38.47%)
21–30	164 (30.89%)	75 (34.88%)	239 (32.04%)
31–40	61 (11.49%)	20 (9.30%)	81 (10.86%)
> 40	33 (6.21%)	7 (3.26%)	40 (5.36%)

MS: multiple sclerosis; SD: standard deviation.

a 530 female probands have known ages of MS onset.

b 214 male probands have known ages of MS onset.

c 744 probands have known ages of MS onset.

d Relatives include parents, full siblings, children, grandparents, aunts/uncles, nieces/nephews, half siblings, and first cousins with complete information on sex, age, or age of death.

Probands in this study (i.e. born 1970 or later) had earlier average ages of onset compared to what the previous work^4^ as expected, given the birth cohort. Within the current birth cohort, female and male probands had comparable average ages of onset. Complete information, including sex, year of birth, present age or age at death (where applicable) was available for 15,955 biological relatives (7915 females; 8040 males) of the MS probands—3069 first-degree (parents, siblings, children), 7294 second-degree (grandparents, aunts/uncles, nieces/nephews, half-siblings), and 5592 third-degree relatives (maternal/paternal first cousins). There were a total of 143 biological relatives with confirmed diagnoses of MS (98 females; 45 males). Age of onset was not available for 70 of the 143 (49%) affected relatives. Table 2 gives a summary of the biological relatives by sex and relation to proband.

**Table 2.** Summary of relatives of MS probands.

Relative category	Female proband		Male proband	
	*N* (affected)	Average age (SD) (95% CI^a^)	*N* (affected)	Average age (SD) (95% CI^a^)
Father	516 (11)	64.31 (9.80) (63.46, 65.16)	204 (5)	63.83 (9.70) (62.50, 65.17)
Mother	526 (14)	62.74 (8.65) (62.00, 63.48)	213 (2)	61.63 ( 8.54) (60.48, 62.78)
F:M ratio of *N*	1.02		1.04	
Brother	372 (2)	36.60 (9.47) (35.64, 37.56)	135 (0)	35.77 (9.96) (34.09, 37.45)
Sister	356 (20)	37.04 (9.66) (36.04, 38.04)	135 (2)	35.81 (8.97) (34.30, 37.32)
F:M ratio of *N*	0.96		1.00	
Son	224 (0)	11.13 (7.02) (10.21, 12.05)	91 (0)	8.35 (6.07) (7.10, 9.60)
Daughter	215 (0)	11.00 (6.37) (10.15, 11.85)	82 (0)	7.71 (5.30) (6.56, 8.86)
F:M ratio of *N*	0.96		0.90	
Nephew	364 (1)	11.23 (7.75) (10.43, 12.03)	127 (0)	11.30 (7.30) (10.03, 12.57)
Niece	348 (0)	11.95 (8.35) (11.07, 12.83)	139 (0)	10.33 (7.64) (9.06, 11.60)
F:M ratio of *N*	0.96		1.09	
Uncle	1349 (10)	60.28 (15.54) (59.45, 61.11)	505 (3)	59.97 (15.13) (58.65, 61.29)
Aunt	1314 (15)	62.31 (13.44) (61.58, 63.04)	507 (1)	60.84 (14.00) (59.62, 62.06)
F:M ratio of *N*	0.97		1.00	
Male first cousin	2136 (6)	36.85 (11.95) (36.34, 37.36)	735 (4)	34.59 (12.17) (33.71, 35.47)
Female first cousin	1991 (20)	36.80 (11.78) (36.28, 37.32)	730 (9)	36.18 (11.91) (35.32, 37.04)
F:M ratio of *N*	0.93		0.99	
Half brother	93 (0)	34.92 (13.06) (32.27, 37.57)	68 (0)	35.96 (17.41) (31.82, 40.10)
Half sister	105 (2)	35.88 (13.74) (33.25, 38.51)	68 (4)	36.71 (15.82) (32.95, 40.47)
F:M ratio of *N*	1.13		1.00	
Grandfather	807 (3)	73.74 (14.79) (72.72, 74.76)	314 (0)	74.00 (13.94) (72.46, 75.54)
Grandmother	848 (8)	78.83 (12.87) (77.96, 79.70)	338 (1)	77.63 (14.56) (76.08, 79.18)
F:M ratio of *N*	1.05		1.08	

SD: standard deviation; CI: confidence interval; F: female; M: male.

a 95% CI: 95% confidence intervals for the average.

There was no difference in the overall sex ratio of biological relatives of female probands compared to male probands. Furthermore, the average ages of relatives of female and male probands were comparable. The overall mean pedigree size was 22 (female probands: 23; male probands: 21).

Crude and age-adjusted RRs for father/mother, brother/sister and uncle/aunt of female and male probands are presented in Table 3. Data for relatives of all probands are given in Table 4.

**Table 3.** Crude and age-adjusted RRs for relatives of female and male probands with MS.

Sex of proband	Relative category	Proportion affected	Crude risk (%)	Age-adjusted risk (%)	95 % CI of age-adjusted risk (%)	LRT statistic^a^
Female	Father	11/516	2.13	2.14	0.89–3.40	0.30
	Mother	14/526	2.66	2.66	1.29–4.04	*p* = 0.58
				F:M ratio = 1.24		
Male	Father	5/204	2.45	2.46	0.33–4.59	1.49
	Mother	2/213	0.94	0.94	0.00–2.14	*p* = 0.22
				F:M ratio = 0.38		
Female	Brother	2/372	0.54	0.66	0.00–1.57	18.04
	Sister	20/356	5.62	6.74	3.88–9.60	*p* = 0.000022
				F:M ratio = 10.21		
Male	Brother	0/135	0.00	0.00	N/A	
	Sister	2/135	1.48	1.86	0.00–4.42	
Female	Uncle	10/1349	0.74	0.78	0.30–1.26	1.02
	Aunt	15/1314	1.14	1.17	0.58–1.76	*p* = 0.31
				F:M ratio = 1.50		
Male	Uncle	3/505	0.59	0.62	0.00–1.31	1.09
	Aunt	1/507	0.20	0.20	0.00–-0.60	*p* = 0.30
				F:M ratio = 0.32		

CI: confidence interval; F: female; M: male.

a LRT statistic: likelihood ratio test statistic (chi-square with one degree of freedom).

**Table 4.** Crude and age-adjusted RRs for relatives of all probands with MS.

Relative category	Proportion affected	Crude risk (%)	Age-adjusted risk (%)	95% CI of age-adjusted risk (%)	LRT statistic^a^
Father	16/720	2.22	2.23	1.15-–3.31	0.0074
Mother	16/739	2.17	2.17	1.12–3.22	*p* = 0.93
			F:M ratio = 0.97		
Brother	2/507	0.39	0.49	0.00–1.16	20.30
Sister	22/491	4.48	5.44	3.23–7.66	*p* = 0.0000066
			F:M ratio = 11.10		
Uncle	13/1854	0.70	0.73	0.34–1.13	0.30
Aunt	16/1821	0.88	0.90	0.46–1.34	*p* = 0.58
			F:M ratio = 1.23		

CI: confidence interval; F: female; M: male.

a LRT statistic: likelihood ratio test statistic (chi-square with one degree of freedom).

The overall age-adjusted RR for sisters (5.44%) was higher than that for brothers (0.49%) of all sex probands taken together (*p* = 0.0000066) with the F:M ratio of the RR being 11.10:1.

The age-adjusted RR for sisters (6.74%) was higher than that for brothers (0.66%) of female probands (*p* = 0.000022) with the F:M ratio of the affected being 10.21:1. The age-adjusted RR for sisters of male probands was 1.86% while no brother was reportedly affected in this study cohort. Age-adjusted risks for mothers (2.66%) and fathers (2.14%) of female probands did not differ (*p* = 0.58); and those for mothers (0.94%) and fathers (2.46%) of male probands also did not differ (*p* = 0.22). No difference was found on the age-adjusted RRs for aunts (1.17%) and uncles (0.78%) of female probands (*p* = 0.31), and those for aunts (0.20%) and uncles (0.62%) of male probands (*p* = 0.30).

Table 5 shows the RR data from the 1988 study^4^ and this study for parents, siblings, and aunts/uncles. Table 6 shows the sex ratios with 95% CIs and the results of comparisons. The F:M ratio of RRs for the mother/father of female probands was 1.86 in 1988 and 1.24 in this study; the F:M ratio of risks for the aunts/uncles of female probands was 1.54 in 1988 and 1.50 in this study. These ratios were comparable. However, the F:M ratio of RRs for sisters/brothers of female probands was 2.49 in 1988 and increased to 10.21:1 in this study.

**Table 5.** Age-adjusted risks for relatives of MS probands from the 1988 study and this study.

Female probands				
Relationship to proband	1988 study^a^		2019, this study	
	Proportion affected	Age-adjusted risk (%)	Proportion affected	Age-adjusted risk (%)
Mother	14/383	3.71	14/526	2.66
Father	6/303	2.00 (F:M ratio = 1.86)	11/516	2.14 (F:M ratio = 1.24)
Parent	20/686	2.95	25/1042	2.41
Sister	25/608	5.65	20/356	6.74
Brother	10/612	2.27 (F:M ratio = 2.49)	2/372	0.66 (F:M ratio = 10.21)
Sibling	35/1220	3.97	22/728	3.66
Aunt	15/674	1.88	15/1314	1.17
Uncle	8/817	1.22 (F:M ratio = 1.54)	10/1349	0.78 (F:M ratio = 1.50)
Aunt/uncle	23/1491	1.59	25/2663	0.97
Male probands				
Mother	7/184	3.84	2/213	0.94
Father	1/128	0.79 (F:M ratio = 4.86)	5/204	2.46 (F:M ratio = 0.32)
Parent	8/312	2.59	7/417	1.68
Sister	9/340	3.46	2/135	1.86
Brother	10/326	4.15 (F:M ratio = 0.83)	0/135	0.00
Sibling	19/666	3.81	2/270	0.93
Aunt	10/310	3.28	1/507	0.20
Uncle	5/250	2.05 (F:M ratio = 1.60)	3/505	0.62 (F:M ratio = 0.32)
Aunt/uncle	15/560	2.68	4/1012	0.41

a Sadovnick et al.^4^

**Table 6.** Comparison of sex ratios for relatives of MS probands from the 1988 study and this study.

Female probands			
Female: Male relative	1988 study^a^	2019, this study	*z*-score^b^ (*p*-value)
	F:M ratio (95% confidence interval)	F:M ratio (95% confidence interval)	
Mother: Father	1.86 (0.73, 4.74)	1.24 (0.57, 2.71)	−0.64 (0.26)
Sister: Brother	2.49 (1.35, 4.59)	10.21 (2.77, 37.67)	−1.92 (0.027)
Aunt: Uncle	1.54 (0.68, 3.51)	1.50 (0.69, 3.27)	0.047 (0.48)
Male probands			
Mother: Father	4.86 (0.61, 38.59)	0.32 (0.075, 1.94)	1.89 (0.029)
Sister: Brother	0.83 (0.39, 1.79)	—	
Aunt: Uncle	1.60 (0.56, 4.57)	0.32 (0.034, 3.02)	−1.27 (0.10)

a Sadovnick et al.^4^

b The significance level of the one-sided *z*-test was set at 0.05.

Using the *z*-test for comparison, the following significant results were found:

1. Siblings of the female probands (*p* = 0.027) suggest an increase in F:M ratio (2.49 with 95% CI: (1.35, 4.59)) from the 1988 study to this study (10.21 with 95% CI: (2.77, 37.67)). Although the two 95% CIs overlap, neither interval contains the other estimate. It must be noted, as seen in Table 5, that the sister risk for female probands increased from 5.65 in 1988 to 6.74 in 2019 and the brother risk decreased from 2.27 in 1988 to 0.66 in 2019.
2. Parents of the male probands (*p* = 0.029) suggest a decrease in F:M ratio (4.86 with 95% CI: (0.61,38.59)) from the 1988 study to this study (0.32 with 95% CI: (0.075, 1.94)). Although the two 95% CIs are overlapped, neither interval contains the other estimate. The parental risk for parents of male probands decreased from 2.59 in 1988 to 1.68 in 2019.

## Discussion

The analyses focused on parents, aunts/uncles, and siblings of probands to maximize completeness of information and to minimize age correction. As of this study’s cut-off date, no child of a proband has been diagnosed with MS. Even though a large number of probands were included in this study, the small numerators in different relation categories of male probands result in many CIs include the value of zero, and no difference in RRs was found between the F and M relatives.

It is recognized that both genetic sharing and environmental factors are important in determining RRs. A higher F:M ratio in RRs in more recently born birth cohorts are more readily explained by environmental differences (potentially modifiable) rather than genetic ones as the latter do not change within populations over mere decades. In our study, we found changes in the F:M ratio of RRs for sisters/brothers of female probands over time. This study did not find any sex bias with respect to having information about biological family members, that is, male probands were as informative as female probands. Therefore, our results provide evidence that environmental factors substantially influence RR data for MS susceptibility, which has important implications.

The impact of information bias is always a consideration. In this study, it is noted that no contributing information (age, health, age at death) is available for 19/746 potential fathers. This represents 2.5% of fathers. A review of the data shows that these fathers lost complete contact with the child as did the fathers’ extended families. This is not unexpected since data from Statistics Canada indicate that 12.8% of Canadian children live in fatherless households, that is, no direct contact with father.^20^

Case capture can never be 100%, but given the purpose of the paper, to compare temporal changes in RR, the critical factor is that the two comparison populations (1988 paper;^4^ this paper) are taken from the same source (UBC MS Clinic). This is as comparable as possible.

Previous work using the UBC MS Clinic data have not shown any systematic differences between included and excluded cases except that we deliberately excluded adoptees in studies of RRs. Again, based on numerous publications from the UBC MS Clinic alone or in combination from other Canadian MS Clinics, BC data are representative of the Canadian population with the exception of First Nations.

As expected, probands in this study (i.e. born 1970 or later) had earlier average ages of onset compared to our previous work^4^ thus making the maximum onset age 49 years or less. This may be viewed as a potential limitation of the study but it is important to note that a recent meta-analysis of late onset MS (LOMS—defined as MS onset at or after the age of 50 years old) found that this totaled only about 5.01% (95% CI 3.78–6.57) of the total MS population.^21^ It has also been reported that the familial risk of MS does not change with age of onset.^22^

The major findings in this study are that there appears to be a decrease in familial risk for all first-degree relatives with the exceptions of biological sisters of female probands and biological fathers of male probands when compared to our previous work.^4^ Familial risk for biological relatives includes both genetic and shared environmental factors. It appears that there is an increase in MS risk due to environmental factors in later life (i.e. not shared family environment) as evident by the decrease in familial risk from the original study.^4^ Genetic factors cannot explain the decreases in MS familial risk, thus, environmental exposures in genetically predisposed individuals might be driving the MS risk.

A recent review^23^ suggests various environmental risk factors for MS with the most replicable to date being hypovitaminosis D, obesity, Epstein–Barr virus (EBV), and smoking.^23^

Significant sex differences in vitamin D metabolism were observed in a case–control study. Women with MS had significantly higher plasma 25-hydroxyvitamin D (25(OH)D) and 1,25-dihydroxyvitamin D3 (1,25(OH)2D3—active form of vitamin D) concentrations than men with MS.^24^ Sex differences in vitamin D metabolism have also been observed in animal research, with dietary vitamin D delaying the onset and severity of the disease in female but not male mice with encephalomyelitis.^25^ Obesity may also play a role not only via alterations in vitamin D bioavailability but also through other mechanisms as outlined below.

There has been a steady increase in the prevalence of obesity in Canadian adults over the past four decades, increasing from 10% in the 1970/1972 to 26% in the 2009/2011.^26^ Strong evidence supports childhood and adolescent obesity as significant risk factors for MS susceptibility. This association has been largely confirmed in females, while evidence in males is mixed.^27^

Obesity is characterized by a chronic, low-grade inflammatory response^28^ and promotes autoimmunity through a variety of mechanisms including secretion of adipokines.^29^ Studies on the effect of excess body fat on the abundances of different bacteria taxa in the gut generally show alterations in the gastrointestinal microbiota with effects on inflammation, insulin resistance, deposition of energy in fat stores.^30^

EBV appears to be the most often identified virus related to all types of MS (pediatric, relapsing-remitting, chronic progressive).^23,31^ Symptomatic EBV (infectious mononucleosis) has been reported to increase the risk for MS and in contrast, EBV negativity may decrease the risk. EBV may also be involved in MS relapses. The exact mechanism(s) is unknown but hopefully more will be learned by interventional studies that eliminate and/or alter EBV-infected memory B cells.^31^

Cigarette smoking is a recognized risk factor for MS.^23,32–34^ A recent systematic review of the literature on MS and smoking used Hill’s criteria^35^ and concluded a causal role for both MS etiology and progression.

In conclusion, in a cohort of people with MS born in 1970 or since, genetic predisposition to MS likely explains part of the disease risk for the general population as well as within families. Sex and environmental factors may also be contributors, which could have implications as some of the environmental factors can potentially be modified. Updated recurrence risk data showing increased F:M ratio in some relationships can be taken into account in genetic counseling as well as in interpreting data from family, molecular genetic, pharmacology, and natural history studies.

## References

[1] Esposito F Guaschino C Sorosina M , et al. Impact of MS genetic loci on familial aggregation, clinical phenotype, and disease prediction. Neurol Neuroimmunol Neuroinflamm 2015; 2(4): e129. 26185776 10.1212/NXI.0000000000000129 PMC4503410

[2] Bijanzadeh M. The recurrence risk of genetic complex diseases. J Res Med Sci 2017; 22: 32. 28461818 10.4103/1735-1995.202143 PMC5390543

[3] https://www.yourgenome.org/search/results/common complex disorders (accessed 29 January 2020).

[4] Sadovnick AD Baird PA Ward RH. Multiple sclerosis: Updated risks for relatives. Am J Med Genet 1988; 29(3): 533–541. 3376997 10.1002/ajmg.1320290310

[5] Orton SM Herrera BM Yee IM , et al. Sex ratio of multiple sclerosis in Canada: A longitudinal study. Lancet Neurol 2006; 5(11): 932–936. 17052660 10.1016/S1474-4422(06)70581-6

[6] Koch-Henriksen N Thygesen LC Stenager E , et al. Incidence of MS has increased markedly over six decades in Denmark particularly with late onset and in women. Neurology 2018; 90(22): e1954–e1963. 29720546 10.1212/WNL.0000000000005612

[7] Boström I Landtblom AM. Does the changing sex ratio of multiple sclerosis give opportunities for intervention? Acta Neurol Scand 2015; 132(199): 42–45. 10.1111/ane.12430 26046558

[8] Kampman MT Aarseth JH Grytten N , et al. Sex ratio of multiple sclerosis in persons born from 1930 to 1979 and its relation to latitude in Norway. J Neurol 2013; 260(6): 1481–1488. 23292231 10.1007/s00415-012-6814-x

[9] Kotzamani D Panou T Mastorodemos V , et al. Rising incidence of multiple sclerosis in females associated with urbanization. Neurology 2012; 78(22): 1728–1735. 22592376 10.1212/WNL.0b013e31825830a9

[10] Fagnani C Neale MC Nisticò L , et al. Twin studies in multiple sclerosis: A meta-estimation of heritability and environmentality. Mult Scler 2015; 21(11): 1404–1413. 25583848 10.1177/1352458514564492

[11] International Multiple Sclerosis Genetics Consortium. Low-frequency and rare-coding variation contributes to multiple sclerosis risk. Cell 2018; 175: 1679–1687. 30343897 10.1016/j.cell.2018.09.049 PMC6269166

[12] International Multiple Sclerosis Genetics Consortium. Multiple sclerosis genomic map implicates peripheral immune cells and microglia in susceptibility. Science 2019; 365(6460): eaav7188. 10.1126/science.aav7188 PMC7241648 31604244

[13] Briggs F. Unraveling susceptibility to multiple sclerosis. Science 2019; 365(6460): 1383–1384. 31604260 10.1126/science.aay1439

[14] https://www.centreforbrainhealth.ca/clinics/clinics/multiple-sclerosis (accessed 31 July 2020).

[15] Poser CM Paty DW Scheinberg L , et al. New diagnostic criteria for multiple sclerosis: Guidelines for research protocols. Ann Neurol 1983; 13(3): 227–231. 6847134 10.1002/ana.410130302

[16] Thompson AJ Banwell BL Barkhof F , et al. Diagnosis of multiple sclerosis: 2017 revisions of the McDonald criteria. Lancet Neurol 2018; 17(2): 162–173. 29275977 10.1016/S1474-4422(17)30470-2

[17] Brownlee WJ Swanton JK Altmann DR , et al. Earlier and more frequent diagnosis of multiple sclerosis using the McDonald criteria. J Neurol Neurosurg Psychiatry 2015; 86(5): 584–585. 25412872 10.1136/jnnp-2014-308675 PMC4451169

[18] Ebers GC Koopman WJ Hader W , et al. The natural history of multiple sclerosis: A geographically based study: 8: Familial multiple sclerosis. Brain 2000; 123(Pt. 3): 641–649. 10686184 10.1093/brain/123.3.641

[19] Risch N. Estimating morbidity risks with variable age of onset: Review of methods and a maximum likelihood approach. Biometrics 1983; 39(4): 929–939. 6671128

[20] https://www12.statcan.gc.ca/census-recensement/2011/as-sa/98-312-x/98-312-x2011001-eng.cfm (accessed 29 January 2020).

[21] Naseri A Nasiri E Sahraian MA , et al. Clinical features of late-onset multiple sclerosis: A systematic review and meta-analysis. Mult Scler Relat Disord 2021; 50: 102816. 33571792 10.1016/j.msard.2021.102816

[22] Song J Westerlind H McKay KA , et al. Familial risk of early- and late-onset multiple sclerosis: A Swedish nationwide study. J Neurol 2019; 266(2): 481–486. 30578428 10.1007/s00415-018-9163-6 PMC6373346

[23] Jacobs BM Giovannoni G Cuzick J , et al. Systematic review and meta-analysis of the association between Epstein-Barr virus, multiple sclerosis and other risk factors. Mult Scler 2020; 26(11): 1281–1297. 32202208 10.1177/1352458520907901 PMC7543008

[24] Barnes MS Bonham MP Robson PJ , et al. Assessment of 25-hydroxvitamin D and 1,25-dihydroxyvitamin D3 concentrations in male and female multiple sclerosis patients and control volunteers. Mult Scler 2007; 13: 670–672. 17548449 10.1177/1352458506072666

[25] Spach KM Hayes CE. Vitamin D3 confers protection from autoimmune encephalomyelitis only in female mice. J Immunol 2005; 175: 4119–4126. 10.4049/jimmunol.175.6.4119 16148162

[26] Janssen I. The public health burden of obesity in Canada. Can J Diabetes 2013; 37(2): 90–96. 24070798 10.1016/j.jcjd.2013.02.059

[27] Gianfrancesco MA Barcellos LF. Obesity and multiple sclerosis susceptibility: A review. J Neurol Neuromedicine 2016; 1(7): 1–5. 10.29245/2572.942x/2016/7.1064 PMC5156319 27990499

[28] Mraz M Haluzik M. The role of adipose tissue immune cells in obesity and low-grade inflammation. J Endocrinol 2014; 222(3): R113–R127. 25006217 10.1530/JOE-14-0283

[29] Versini M Rosenthal E Jeandel PY , et al. Obesity in autoimmune diseases: Not a passive bystander. Autoimmun Rev 2014; 13(9): 981–1000. 25092612 10.1016/j.autrev.2014.07.001

[30] Ley RE. Obesity and the human microbiome. Curr Opin Gastroenterol 2010; 26(1): 5–11. 19901833 10.1097/MOG.0b013e328333d751

[31] Bar-Or A Pender MP Khanna R , et al. Epstein-Barr virus in multiple sclerosis: Theory and emerging immunotherapies. Trends Mol Med 2020; 26(3): 296–310. 31862243 10.1016/j.molmed.2019.11.003 PMC7106557

[32] Hedström AK Olsson T Alfredsson L. Smoking is a major preventable risk factor for multiple sclerosis. Mult Scler 2016; 22(8): 1021–1026. 26459151 10.1177/1352458515609794

[33] Degelman ML Herman KM. Smoking and multiple sclerosis: A systematic review and meta-analysis using the Bradford Hill criteria for causation. Mult Scler Relat Disord 2017; 17: 207–216. 29055459 10.1016/j.msard.2017.07.020

[34] Rosso M Chitnis T. Association between cigarette smoking and multiple sclerosis: A review. JAMA Neurol 2020; 77: 245–253. 31841592 10.1001/jamaneurol.2019.4271

[35] Hill AB. The environment and disease: Association or causation? 1965. J R Soc Med 2015; 108: 32–37. 25572993 10.1177/0141076814562718 PMC4291332

